# Supplementary material for: Discriminating miRNA Profiles between Endometrioid Well- and Poorly-Differentiated Tumours and Endometrioid and Serous Subtypes of Endometrial Cancers
Source: Int J Mol Sci. 2020 Aug 23;21(17):6071. doi: 10.3390/ijms21176071 (PMC7504607; doi:10.3390/ijms21176071)
Supplement: Supplementary file 1 [file ijms-21-06071-s001.pdf]

Table S1. Target genes of significantly down-regulated miRNAs in more advanced subtypes of endometrial carcinoma.

EC subtypes	Down-regulated miRNAs*	Experimentally validated target genes by reporter assay, Western blot or qPCR
Endometrioid grade 1 vs grade 3	let-7c-5p	*AGO1, BCL2L1, CEBPB, COPS6, COPS8, DICER1, GPS1, HMGA2, HSPA4, IGF1R, IL6, IL10, ITGB3, MAP4K3, MPL, MTOR, MYC, NRAS, NUMB, PBX2, RICTOR, TGFBR1, TNFRSF10B, TRIB2, TRIM71*
	miR-125b-5p	*ABTB1, AHRR, AKT1, ARID3A, ARID3B, BAK1, BBC3, BCL2, BCL2L2, BCL3, BMF, BMPR1B, BTG2, CBFB, CCNJ, CDH5, CDKN2A, CSNK2A1, CYP24A1, DGAT1, DUSP6, E2F2, E2F3, EIF4EBP1, EIF5A2, ENPEP, EPO, EPOR, ERBB2, ERBB3, ETS1, FGFR2, GLI1, GRIN2A, GSS, HK2, HMGA1, HMGA2, ICAM2, IGF2, IKZF2, IKZF3, IKZF4, IL6R, IRF4, KLF13, LACTB, LIF, LIN28A, LIN28B, LIPA, MAN1B1, MAP3K11, MAPK14, MCL1, MEGF9, MMP2, MMP13, MMP26, MUC1, MXD1, NCOR2, NES, NKIRAS2, NTRK3, PCTP, PIAS3, PIGF, PIK3CD, PPP1CA, PRDM1, PRKRA, RAF1, RPS6KA1, SCNN1A, SET, SFRP5, SGPL1, SIRT7, SMAD4, SMO, SPHK1, STARD13, STAT3, TBC1D1, TET2, TNFAIP3, TP53, TP53INP1, VDR, VPS4B, VPS51*
	miR-23b-3p	*ATG12, CA2, CHUK, ETS1, HMGA2, HMGB2, MARCKS, MET, NOTCH1, NOTCH2, PLAU, PRAP1, PRKACB, PTEN, RRAS2, RUNX2, SRC, TAB2, TAB3, TMEM64, TUSC7, VHL, ZEB1*
	miR-99a-5p	*AGO2, AKT1, CTDSPL, FGFR3, FKBP5, HOXA1, GRHL1, IGF1R, MTOR, MTMR3, PPP1CB, RAVER2, SERPINE1, SMARCA5, TRIB2*
Endometrioid vs Serous	let-7g-5p	*AGO1, AKT2, BCL2L1, BMI1, CASP3, CDKN2A, COL1A2, FN1, GAB2, HMGA2, IGF2BP1, IL13, KRAS, MYC, SMAD2, TBC1D9, TGFBR1,THBS1, TNFRSF10B*
	miR-195-5p	*ALOX12, ARL2, ATG14, Bace1, BCL2, BCL2L2, BIRC5, BTRC, CAB39, CBX4, CCL4, CCND1, CCND3, CCNE1, CDC25A, CDC42, CDK4, CDK6, CGNL1, CHEK1, CHUK, DICER1, E2F3, ELN, FASN, HMGA1, INSR, KIF21A, KRT7, MBD1, MYB, NKD1, PDIA6, PHACTR2, RAF1, RASGEF1B, RET, RPS6KB1, RUNX2, SNX16, SLC2A3, SMAD7, STRADB, TAB3, TBCCD1, TRIP10, TUFT1, VEGFA, WEE1, WNT7A*
	miR-34a-5p	*ABLIM1, ACSL1, ACSL4, AIM1, AR, AREG, ARHGDIB, AXL, AXIN2, B4GALT5, BAX, BCL2, BIRC5, BMP7, CCL22, CCND1, CCND3, CCNE2, CD24, CD44, CDC25A, CDK4, CDK6, CDKN2A, CDKN2C, CEBPB, CLOCK, CSF1R, CYBB, DGUOK, DLL1, DOCK3, E2F1, E2F3, EPHA5, FOSL1, FOXP1, FUT8, GALNT7, GAS1, GFRA3, GRM7, HDAC1, HNF4A, HOTAIR, HPRT1, IL6R, IFNB1, IMPA1, IMPDH2, INHBB, JAG1, KCNH1, KDM4A, KIT, KLB, KLF4, KLF12, L1CAM, LEF1, LDHA, MAGEA2, MAGEA3, MAGEA6, MAGEA12, MAP2K1, MAP3K9, MARCH5, MDM4, MET, MMP7, MTA2, MYB, MYC, MYCN, NAMPT, NANOG, NCOA1, NOTCH1, NOTCH2,* NUMB, *PCBP2, PDGFRA, PDGFRB, PEA15, POU5F1, PPP1R10, RICTOR, RNF169, SIRT1, SIRT7, SNAI1, SOX2, SPI1, SRC, STX1A, SYT1, TCF7, TGIF2, TOM1, TP53, ULBP2, VAMP2, VEGFA, WNT1, ZAP70, YY1*
	miR-497-5p	*BCL2, BIRC5, BTRC, CCND3, CCNE1, CDC25A, CDK4, CHEK1, DICER1, E2F3, EIF4E, HDGF, HSPA4L, IGF1R, KCNN4, MAP2K1, RAF1, RUNX2, SMAD3, WEE1, WNT7A*

*miRNAs were down-regulated in both evaluated subtypes compared to controls and statistical difference between expression of compared subtypes was found. Data were adopted from miRTarBase.

Table S2. Top ten KEGG pathways comparing 44 genes involved in endometrial carcinoma.

Pathway	Term	Count	%	P-Value	FDR
hsa05213	Endometrial cancer	44	100.00	1.10E-97	2.84E-100
hsa05200	Pathways in cancer	41	93.20	2.84E-47	3.29E-44
hsa05215	Prostate cancer	35	79.50	1.53E-59	1.77E-56
hsa05210	Colorectal cancer	31	70.45	3.28E-55	3.80E-52
hsa05223	Non-small cell lung cancer	30	68.20	2.65E-54	3.06E-51
hsa05205	Proteoglycans in cancer	30	68.18	1.91E-35	2.21E-32
hsa0415	PI3K-Akt signaling pathway	30	68.18	2.60E-28	3.01E-25
hsa05221	Acute myeloid leukemia	28	63.60	5.11E-49	5.91E-46
hsa04012	ErbB signaling pathway	28	63.60	1.50E-42	1.73E-39
hsa04068	FoxO signaling pathway	27	61.36	8.50E-35	9.83E-32

Pathways search was performed with David 6.7 online tool.
